# The Prognostic Value of ^18^F-FDG PET Imaging at Staging in Patients with Malignant Pleural Mesothelioma: A Literature Review

**DOI:** 10.3390/jcm11010033

**Published:** 2021-12-22

**Authors:** Silvia Taralli, Romina Grazia Giancipoli, Carmelo Caldarella, Valentina Scolozzi, Sara Ricciardi, Giuseppe Cardillo, Maria Lucia Calcagni

**Affiliations:** 1 UOC di Medicina Nucleare, Radioterapia Oncologica ed Ematologia, Dipartimento di Diagnostica per Immagini, Fondazione Policlinico Universitario Agostino Gemelli IRCCS, 00168 Rome, Italy; silvia.taralli@hotmail.it (S.T.); romina.giancipoli@gmail.com (R.G.G.); valentina.scolozzi@gmail.com (V.S.); marialucia.calcagni@unicatt.it (M.L.C.); 2 Department of Cardiothoracic Surgery, S. Orsola-Malpighi University Hospital, 40138 Bologna, Italy; ricciardi.sara87@gmail.com; 3 Unit of Thoracic Surgery, Azienda Ospedaliera San Camillo Forlanini, 00152 Rome, Italy; gcardillo@scamilloforlanini.rm.it; 4 Dipartimento Universitario di Scienze Radiologiche ed Ematologiche, Università Cattolica del Sacro Cuore, 00168 Rome, Italy

**Keywords:** mesothelioma, pleural, PET/CT, ^18^F-FDG, prognosis

## Abstract

Malignant pleural mesothelioma (MPM) is an aggressive malignancy, frequently diagnosed at locally-advanced/metastatic stages. Due to a very poor prognosis and limited treatment options, the need to identify new prognostic markers represents a great clinical challenge. The prognostic role of metabolic information derived from Positron Emission Tomography (PET) with ^18^F-Fluoro-deoxy-glucose (^18^F-FDG) has been investigated in different MPM settings, however with no definitive consensus. In this comprehensive review, the prognostic value of FDG-PET imaging exclusively performed at staging in MPM patients was evaluated, conducting a literature search on PubMed/MEDLINE from 2010 to 2020. From the 19 selected studies, despite heterogeneity in several aspects, staging FDG-PET imaging emerges as a valuable prognostic biomarker, with higher tumor uptake predictive of worse prognosis, and with volumetric metabolic parameters like Metabolic Tumor Volume, (MTV) and Total Lesion Glycolisis (TLG) performing better than SUVmax. However, PET uptake parameters were not always confirmed as independent prognostic factors, especially in patients previously treated with pleurodesis and with a non-epithelioid histotype. Future prospective studies in larger and clinically homogeneous populations, and using more standardized methods of PET images analysis, are needed to further validate the value of staging FDG-PET in the prognostic MPM stratification, with a potential impact on better patient-tailored treatment planning, in the perspective of personalized medicine.

## 1. Introduction

Malignant pleural mesothelioma (MPM) is the most common primary neoplasm of the pleura, arising from pleural mesothelial cells, and it is usually related to occupational or environmental asbestos exposure [1]. Although MPM is considered a rare malignancy, with about 20 cases per million per year in Europe [2,3], its worldwide incidence is increasing in recent years due to widespread asbestos exposure over the last decades, a mean 20–40 years latency from exposure to tumor development, and lack of restrictions about the use of asbestos in many countries [1,4]. MPM is a highly aggressive tumor with a very poor prognosis, with most patients presenting at diagnosis with locally-advanced or metastatic disease [5], and with a median survival of 9–17 months, or even lower (4–8 months) if untreated [6]. Despite, at present, there being no widely accepted standard of care, treatment strategy is mainly determined by disease stage (I-II vs. III-IV), histological type (epithelioid vs. non-epithelioid), and patient’s performance status. Generally, multimodality therapy (combining surgery with either neoadjuvant and/or adjuvant treatment that includes chemotherapy and radiotherapy) with curative intent is considered in selected patients judged medically operable, with limited stages and epithelioid subtype, whereas the remaining cases (unfortunately representing the majority of patients) are candidates to systemic chemotherapy with pemetrexed and platinum-based agents (+/− radiotherapy) or palliative-best supportive care [4,7,8,9]. Nevertheless, regardless of the therapeutic strategy, the survival benefits of the current treatment options remain unsatisfactory, even in the case of multimodality therapy that has improved survival in select patients, but with inter-individual variability in response [10,11]. Therefore, the prognostic stratification of MPM patients remains a great challenge for clinicians, both in aiming for a better patient-tailored treatment strategy and in the perspectives of new therapeutic frontiers and patients’ enrolment into prospective clinical trials.

Currently recognized clinical-pathological predictors of worse prognosis are mainly represented by male gender, non-epithelioid histology, advanced stage, ECOG performance status > 0, and an age older than 75 years [12,13]. However, the need for additional and stronger prognostic markers is increasingly emerging in recent years, especially assuming that more complex and patient-specific tumor characteristics among different MPM patients may impact on the clinical course of disease much more than these conventional prognostic indexes. With this perspective, the greatest research interest has been focused on the potential predictive role of biomarkers able to reflect intrinsic biological characteristics of MPM cells, ranging from serum, tissue, molecular, and genetic markers to metabolic features derived from Positron Emission Tomography/Computed Tomography (PET/CT) with ^18^F-Fluoro-deoxy-glucose (^18^F-FDG) [14,15,16].

Particularly, metabolic information provided by ^18^F-FDG PET/CT, which is a currently used functional imaging modality in MPM patients for staging, restaging, and treatment response assessment [4,17,18,19], has been widely investigated as a potential prognostic biomarker, considering the MPM metabolic activity as a marker of biological aggressiveness. Indeed, stated that an increased tumor ^18^F-FDG uptake reflects an increased tumor glucose metabolism needed for cellular proliferation, a higher intensity of tracer uptake at PET imaging is generally observed in more aggressive and prognostically unfavourable tumors. From literature studies evaluating the prognostic role of PET imaging [20,21,22,23], the most analyzed prognostic parameter was the maximum standardized uptake value (SUVmax), the commonest semiquantitative measure used in clinical practice to quantify ^18^F-FDG tumor activity. Few and more recent studies have investigated the potential predictive role of PET volumetric parameters, such as the metabolic tumor volume (MTV) and the total lesion glycolysis (TLG), that represent more advanced semiquantitative PET measures able to better reflect the intrinsic heterogeneity of tumor lesions [24,25]. The prognostic value of PET imaging was assessed in different MPM clinical settings, with PET performed at staging in MPM patients at first diagnosis, at restaging in patients with disease recurrence, or for treatment response evaluation in patients submitted to chemotherapy. From literature evidence, a predictive prognostic value of ^18^F-FDG PET imaging has been demonstrated by several studies, with high tumor ^18^F-FDG uptake at staging or restaging associated with poor outcome [20,21,22,26], and with metabolic response/decrease in tumor activity after chemotherapy predictive of a less aggressive disease evolution [27,28,29,30,31]. However, controversial evidence exists, with other studies that did not report any correlation between MPM metabolic activity and survival, especially when SUVmax was evaluated [32,33,34,35,36]. In particular, no definitive consensus has been established about the prognostic role of ^18^F-FDG PET imaging performed for staging, also with discordant results when both SUVmax and volumetric PET parameters were evaluated [34,35,37]. Moreover, a comprehensive and updated review exclusively focused on the PET staging setting (i.e., MPM patients not submitted to previous treatments) is still lacking.

Therefore, the aim of our study was to review the current literature about the prognostic role of staging ^18^F-FDG PET imaging in patients with MPM.

## 2. Materials and Methods

### 2.1. Literature Search Strategy

A comprehensive computer literature search of PubMed/MEDLINE database over the last 10 years (January 2010–December 2020) was conducted in order to find relevant published articles on the prognostic role of ^18^F-FDG PET imaging performed at staging in patients with MPM at first diagnosis. A search algorithm based on the combination of the terms (“PET” OR “positron emission tomography”) AND ((“mesothelioma” OR “mesothelial” AND “pleural”)) was used. To expand our search, references of the retrieved articles were also screened for additional studies. Titles and abstracts of the retrieved studies were screened independently by two researchers (ST and RGG).

### 2.2. Inclusion and Exclusion Criteria

Only articles written in the English language and studies conducted on human subjects were considered potentially eligible for inclusion. An initial selection was performed excluding (a) articles not within the field of interest of this review (e.g., articles focusing on PET performance for MPM diagnosis, evaluating MPM patients at recurrence, disease other than MPM…); (b) review articles and meta-analyses, editorials, letters, commentaries, and conference proceedings; (c) case reports and case series; (d) pre-clinical studies; and (e) articles with fewer than 10 patients. The same two researchers then independently reviewed the full-text version of the selected articles for final eligibility and conducted data extraction. Only articles that considered MPM patients at first diagnosis, who performed ^18^F-FDG PET imaging for staging, regardless of disease stage, and with available data for prognostic analysis, were finally included. Articles including MPM patients who performed a first ^18^F-FDG PET imaging for staging (before any therapy) and a second PET scan for treatment response evaluation (after therapy) were also included, if prognostic analysis of pre-treatment PET data was available. In the case of possible duplicate publication (data overlap between two or more studies from the same group), only the most complete article was retained for the purposes of this review. Disagreements were resolved by consensus, with two additional researchers (CC and VS) participating in the discussion.

### 2.3. Data Extraction and Analysis

For each included article, information was collected about basic study characteristics (authors, year of publication, journal, country, and study design), patients’ characteristics (number of patients with MPM, number of patients with MPM evaluated with PET, gender, age, MPM histological subtype, stage of disease, previous pleurodesis, and treatment strategy after staging PET), and PET technical aspects (PET modality, injected tracer activity, scan delay defined as time interval between tracer injection and image acquisition, dual time imaging, field of view, semiquantitative image analysis, and tumor contouring method applied to PET images, if applicable/available). In order to assess the prognostic value of ^18^F-FDG PET imaging, the following prognostic data were collected for each included study: PET prognostic value (yes/no), PET-derived prognostic parameters (including optimal cut-off values, when clearly available), and prognostic endpoints. In the case of articles including a second PET scan after treatment, additional information about post-treatment PET imaging (treatment type, PET timing after treatment) was also recorded.

## 3. Results

### 3.1. Literature Research

**Figure 1:**
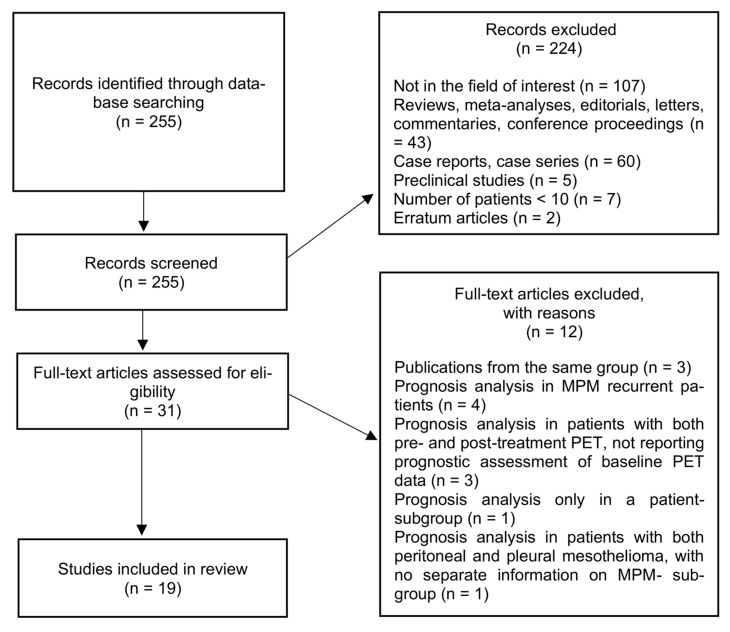
Studies’ inclusion flowchart. MPM, malignant pleural mesothelioma.

The research flowchart is presented in Figure 1. From initial literature research, 255 studies were retrieved. After screening titles and abstracts, 224 studies were excluded. Analyzing the full-text version of the remaining 31 studies, there were 12 excluded (4 articles including patients with MPM recurrence); 3 articles from the same referring center; 3 articles including MPM patients with both baseline (pre-treatment) and post-treatment PET, but not reporting prognostic analysis of baseline PET data; 1study with a prognostic analysis performed only in a subgroup population; and 1 article including patients with both peritoneal and pleural mesothelioma, with no separate information on MPM subgroup). Finally, 19 studies were included and analyzed in this review [29,32,33,34,35,37,38,39,40,41,42,43,44,45,46,47,48,49,50].

### 3.2. Study Characteristics

Main clinical characteristics of all the included studies are summarized in Table 1. Regarding the study design, all except two studies [34,46] were retrospective. When considering study population, a heterogeneous sample size was observed, ranging from 23 to 188 patients, with only 5/19 studies enrolling more than 100 patients. From a total of 1552 MPM enrolled patients, 1472 patients were finally considered in our review, due to the availability of data for PET prognostic assessment. Regarding patients’ characteristics, male gender was highly prevalent in almost all studies; study populations homogeneously presented a mean or median age < 75 years; information on MPM histological subtype was reported in all studies, with epithelioid histology the predominant one; and almost all studies enrolled MPM patients presenting with any stages of disease (from I to IV), although patients with locally advanced and metastatic disease were numerically predominant. In this context, selected patients with a homogeneous clinical staging were exclusively analyzed only in three papers [29,45,50]. Regarding clinical indications of PET exams, in all the included studies, ^18^F-FDG PET imaging was performed for staging purpose (i.e., in MPM patients at first diagnosis) according to the research topic and inclusion criteria of our review: in 17/19 studies, ^18^F-FDG PET imaging was exclusively performed at baseline (before any therapy) [32,33,34,35,37,38,39,40,41,42,43,44,45,47,48,49,50]; in the remaining 2/19 studies [29,46], a second PET scan after systemic therapy was performed for treatment response assessment. Information on talc pleurodesis performed before PET scans was available in 13/19 studies; almost half of these studies (6/13) included a cohort of patients submitted to previous talc pleurodesis, ranging from 30% to 50% [29,32,34,41,44,46].

### 3.3. Methodological Aspects

Methodological aspects of the 17 studies exclusively performing ^18^F-FDG PET imaging at baseline are reported in Table 2. Methodological characteristics of the 2 studies performing ^18^F-FDG PET imaging both at baseline and after treatment are reported in Table 3. Overall, in 16/19 studies the applied PET modality was an integrated PET/CT in all enrolled patients, mostly performing a coregistered low-dose unenhanced CT scan; in two studies PET alone imaging was reported [34,44], whereas another study performed PET alone in a portion of patients included in the older investigational period [29]. Scan delay was about 60 min after ^18^F-FDG intravenous injection in almost all studies, with PET imaging performed at a later time (90 min post-injection) in only three papers [34,37,46]; a dual time imaging was acquired only in the study by Abe Y et al. [38], with a standard scan at 60 min post-injection, followed by an additional delayed scan at 120 min. The prevalent field of view (FOV) of PET acquisition was from the vertex or skull base to the thighs (as the standard FOV in oncological PET), with only two studies extending the lower scan limit to the feet [40,47]. The prognostic value of PET imaging, in terms of overall survival (OS) or progression-free survival (PFS), was assessed by a semiquantitative analysis of tumor ^18^F-FDG uptake in all studies, extracting different PET tumor parameters to be tested as prognostic predictors: SUV-based semiquantitative parameters were considered in all studies, whereas metabolic volumetric parameters were additionally analyzed in 8/19 studies [29,34,35,37,45,46,47,50]. In particular, among SUV-based parameters, tumor SUVmax (i.e., the maximum ^18^F-FDG activity concentration within the tumor lesion, measured in the so called “hottest” pixel) was the most frequent and, in most cases, the only measure considered [33,38,40,41,42,43,44,48,49]; other articles have additionally evaluated tumor SUVpeak (i.e., the mean uptake value in a 1 cm^3^ volume of interest around the hottest pixel) and/or tumor SUVmean (i.e., the mean tumoral uptake value). Both early and delayed SUVmax (at PET scan acquired at 60- and 120-min post-injection, respectively) were considered by Abe et al. [38]. As additional quantitative parameters, Kaira et al. [39] also evaluated the prognostic role of tumor-to-mediastinum ratio (tumor SUVpeak/mediastinal SUVmean), using the mediastinum blood-pool activity as background reference; another study by Ozmen et al. [45] evaluated the prognostic role of both bone marrow–liver ratio (bone marrow SUVmean/liver SUVmean), and of bone marrow (BM) activity alone, according to a visual image analysis (normal BM distribution: score 0; increased ^18^F-FDG uptake on pelvis and lumbar spine: score 1; additionally increased ^18^F-FDG uptake on rib, humerus and proximal femur: score 2). Regarding the analysis of the PET volumetric parameters, both the metabolic tumor volume (MTV that represents, within the tumor volume of interest, the volume of the most metabolically active areas, defined as above a certain threshold of activity) and the total lesion glycolysis (TLG, defined as the product of tumor SUVmean and MTV) were evaluated as prognostic factors in 5/8 studies [35,37,45,46,47]; only metabolic tumor volume was assessed in the study by Pavic et al. (defined as MTV) and in the study by Nowak et al. (defined as total glycolytic volume, TGV) [34,50]; only TLG was considered by Zucali et al. [29]. Regarding the methodology used to practically define and calculate the metabolic tumor volume on PET images, a great heterogeneity was observed among these studies, with different applied methods of tumor contouring/segmentation. Indeed, tumor volume of interest (VOI) was calculated in some studies based on a fixed percentage of the maximal activity in the tumor, with threshold set at a different percentage of SUVmax ranging from 20% to 70% [37,45,50]; in another case it was based on a fixed SUV threshold, with the VOI including all the voxels with a SUV > 2.5 within both lungs, excluding sites of physiological uptake [46]; in one study it was based on a background-level threshold, with liver activity as reference [35]; in other cases VOI were defined by iterative algorithms with adaptive threshold [34,47]; and in one study both iterative algorithm and liver-based threshold contouring were used [29]. Beside SUV-based parameters (SUVmax and SUVmean) and MTV, one recent study by Pavic et al. [50] also evaluated the prognostic role of radiomics application, an advanced image analysis technique used for quantifying PET tumor uptake. The authors retrospectively evaluated the prognostic role of 780 quantitative parameters (radiomics features) extracted from PET/CT performed at staging in 72 MPM patients then treated with curative surgery, with patients equally split into a training and a validation cohort (36 patients each). Regarding the two studies performing ^18^F-FDG PET imaging both at baseline and after systemic treatment, both studies evaluated the baseline SUVmax and the baseline TLG, with Hall et al. [46] also assessing the baseline MTV. Moreover, the percentage changes in SUVmax and in volumetric parameters between baseline and post-treatment PET exams were also analyzed as prognostic factors. In both cases, the second PET scan was performed after two cycles of chemotherapy [29,46], which consisted of up-front pemetrexed-based chemotherapy in one study [29] and of first-line pemetrexed plus platinum-based regimens in the other [46]. Finally, in the 17 studies exclusively performing ^18^F-FDG PET imaging at baseline, data on the subsequent treatment strategy were available in all but one study (Table 2). Heterogeneity regarding treatment modalities (multimodality therapy, chemotherapy alone, radiotherapy alone, chemo-radiotherapy, best supportive care…) was evident both among different studies and also in the single study; a patient-group surgically treated (surgery alone or combined in a multimodality approach) was present in almost all study populations.

### 3.4. PET Prognostic Value

Results of all 19 studies evaluating the prognostic role of ^18^F-FDG PET imaging in MPM patients are detailed in Table 4. Overall, metabolic tumor information derived from staging ^18^F-FDG PET resulted as a prognostic biomarker in 17/19 studies [29,34,35,37,38,39,40,41,42,43,44,45,46,47,48,49,50], with higher tumor uptake predictive of a worse prognosis, whereas no PET predictive role was found in the two studies by Genestreti et al. [32] and Özyürek et al. [33]. In each of these 17 studies, for any PET uptake parameter (SUVmax and/or MTV and/or TLG) resulted of prognostic relevance, a cut-off value was identified for MPM patients’ stratification, with patients with tumor uptake values above the cut-off showing a worse prognosis than those with lower values. Widely heterogeneous optimal cut-off values were reported, mainly identified by ROC curve analysis or arbitrarily defined as the median value observed in each study population: SUVmax, MTV, and TLG cut-offs for prognostic prediction ranged from 3.5 to 10.1, from 112 to 755 cm^3^ and from 525 to 2914, respectively. In detail, 9/17 studies evaluated SUV-based parameters only; tumor SUVmax resulted as the only prognostic factor when also SUVmean and SUVpeak were tested [38,39,40,41,42,43,44,48,49]. The remaining 8/17 studies considered both SUV-based and volumetric parameters (MTV and TLG) [29,34,35,37,45,46,47,50]: volumetric parameters showed a better prognostic value than SUV measure in 5/8 cases [34,35,37,47,50], with SUVmax of borderline significance and at univariate analysis only (in the studies by Nowak et al. and Klabatsa et al.), or significant at univariate analysis only (as reported by Doi et al.), or with totally no prognostic prediction power (as found by Lee et al. and Pavic et al.); SUVmax remained the only significant prognostic factor at multivariate analysis (if histology variable was excluded) in one study, when compared to MTV and TLG [46]; all PET parameters were significantly associated with prognosis in the remaining two studies [29,45]. When, in the same study, both volumetric parameters (MTV and TLG) were evaluated, a not univocal predominance of one over the other was found: MTV and TLG showed an equal association with survival in 3/5 studies [35,37,46]; only TLG remained significant at multivariate analysis in one study [47]; only MTV remained significant at multivariate analysis in another study [45].

Regarding the two studies by Kaira et al. [39] and Ozmen et al. [45], which tested new metabolic parameters beside the commonest PET semiquantitative ones, a prognostic significance was found for the tumor-to-mediastinum (T/M) ratio, with higher values associated with shorter survival (T/M cut-off: 4.23), and for bone marrow (BM) visual activity, with diffuse BM hypermetabolism (BM score > 2) resulting as an independent negative prognostic determinant in terms of survival.

From the only study that evaluated the innovative application of radiomics analysis, Pavic et al. [50] reported that radiomic MPM features extracted from PET images show a good prognostic power especially for PFS, and that radiomic model seemed to perform better than common semiquantitative parameters. Indeed, the metabolic tumor volume (VOI defined according to six different SUVmax%-thresholds) showed no prognostic performance for PFS and was prognostic for OS, but at a lower level than the PET radiomics-based PFS model, whereas a simple model based on SUVmax or SUVmean was not prognostic for PFS nor OS.

When specifically considering the two studies performing ^18^F-FDG PET imaging both at baseline and after treatment [29,46], all PET parameters derived from staging PET (baseline SUVmax, MTV and TLG) resulted as prognostic predictors in both cases, with higher values associated with a worse outcome. Differently, the percentage changes in SUVmax and in volumetric parameters between baseline and post-treatment PET exams showed a prognostic value only in the study by Zucali et al., whereas no changes in PET measures after treatment were related to survival in the study by Hall et al.

Stated the overall demonstrated PET prognostic value, when analyzing in more detail the specific contribute of the PET parameters, they appeared predictive of prognosis only “partially” or “conditionally” to the presence/absence of other factors in 7/17 studies: (a) Nowak et al. [34] showed that total glycolytic volume was a significant predictor of survival only in MPM patients with non-sarcomatoid histology, whereas in patients with sarcomatoid MPM histology resulted the strongest prognostic factor and the addition of other variables was not contributory; (b) Klabatsa et al. [37] reported that MTV and TLG were associated with OS on univariable analysis, whereas on multivariable analysis TLG showed an association with OS, although with borderline statistical significance; (c) in the study by Koyuncu et al. [42], at univariate analysis patients with tumor SUVmax < 8 showed a longer survival time, but SUVmax was not confirmed as an independent prognostic factor at multivariate analysis; d) Billè et al. [44] reported that patients with a tumor SUVmax < 8.1 (versus those with SUVmax > 8.1) showed a better median OS, but with SUVmax significant only in univariate analysis; (e) Zucali et al. [29] reported different prognostic results of PET parameters between patients submitted or not submitted to previous pleurodesis: differently from baseline SUVmax (showing a significant correlation with PFS and OS in both patients groups), baseline TLG showed a significant correlation with OS only in those treated with pleurodesis, and both SUVmax reduction (ΔSUV ≥ 25%) and TLG reduction (ΔTLG ≥ 30%) after treatment were significantly associated with a reduction in the risk of disease progression and death only in patients not receiving pleurodesis; (f) in the study by Hall et al. [46] baseline SUVmax, MTV and TLG were significant factors for OS at univariate Cox regression analysis, whereas SUVmax only remained significant in multivariate analysis, and only if the histology variable was excluded; and (g) Lim et al. [48] found different prognostic results of SUVmax according to MPM histotype: SUVmax was significantly associated with OS among all patients and in patients with the epithelioid subtype, but not in the non-epithelioid subtype.

Finally, regarding the 2/17 studies without any evidence of a prognostic role of staging PET [32,33], both were retrospective and evaluated the prognostic value of SUV-based parameters only. In details, Genestreti et al. [32] investigated PET data of 27 MPM patients with disease stage I-III and surgical resection performed in 44% of cases: they did not find a statistically significant difference in survival between patients with SUVmax/SUVmean higher or lower than median values (cut-off: SUVmax 4.21 and SUVmean 2.78), although the observed difference in median survival (about 7 months) between patients with high and low tumor uptake was considered clinically important. In the other study, Özyürek et al. [33] investigated 67 MPM patients with disease stage I-IV and a little surgically-treated cohort (4 patients): although the median survival time of patients with tumor SUVmax < 5 was longer than those with SUVmax ≥ 5 (23 vs. 13 months), this difference was not statistically significant.

## 4. Discussion

### 4.1. PET Prognostic Role

Data from our review, despite heterogeneity in several clinical and methodological aspects among the included studies, support the prognostic role of ^18^F-FDG PET imaging performed at staging in MPM patients, with higher tumor uptake predictive of worse prognosis. Underlying biologic mechanisms of ^18^F-FDG uptake can explain this relationship between increased tumor activity and unfavourable prognosis, pointing out the role of MPM metabolic behaviour as a marker of its biological aggressiveness. Indeed, as general cellular uptake mechanism, ^18^F-FDG is internalized by glucose transporters (GLUTs), phosphorylated by hexokinase, and so trapped within cells. In MPM cells, it has been demonstrated that increased ^18^F-FDG uptake is associated not only with an over-expression of GLUT-1 and hexokinase I, both reflecting an increased glucose metabolism needed for tumor growth, but also with an up-regulation of tumoral cells factors related to angiogenesis (such as vascular endothelial growth factor, VEGF), hypoxia (hypoxia-inducible factor-1 alpha—HIF-1a—cell proliferation (Ki-67 index), and cell cycle regulation (such as p53), which are well-known to be associated with a more aggressive behaviour and worse prognoses [39]. Especially, hypoxia is recognized as a marker of poor prognosis due to association with tumor aggressiveness and cellular resistance to chemotherapy and radiation treatment, with hypoxic areas being more prominent in the centre of tumor bulky masses, due to relatively poorer vascularization. In this context, a complex relationship among increased ^18^F-FDG uptake, tumor hypoxia, and neoangiogenesis has been observed: hypoxia-inducible factor-1 alpha (HIF-1a), which is upregulated under hypoxic state, is able to induce an up-regulation of GLUT-1 expression (so increasing the energetic supply of tumor cells) and may also induce the expression of the vascular-endothelial growth factor (VEGF), which promotes tumor cell survival and growth stimulating tumor neoangiogenesis (so increasing the tumor blood supply). In addition, tumor proliferation is considered one of the major factors associated with prognosis, being that uncontrolled proliferation is a key characteristic of malignant tumors. In this context, it is well-recognized the relationship between ^18^F-FDG metabolism and tumor growth, with a higher tracer uptake observed in more aggressive and rapidly proliferating tumors.

**Figure 2:**
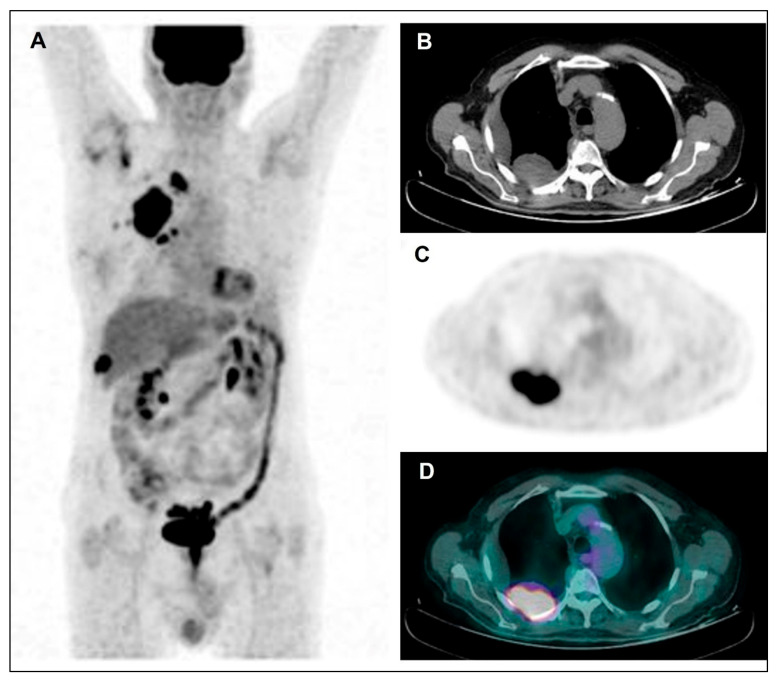
Maximum Intensity Projection—MIP (**A**), coregistered low-dose CT (**B**), ^18^F-FDG PET (**C**), and fused PET/CT (**D**). Images in a male patient with multiple right pleural thickenings showing high tracer uptake along with pleural effusion: the largest and most active lesion is located in the posterior side of costal pleura, as shown in the figure. Afterwards, the patient underwent VATS and pleural sampling with diagnosis of diffuse bi-phasic MPM.

**Figure 3:**
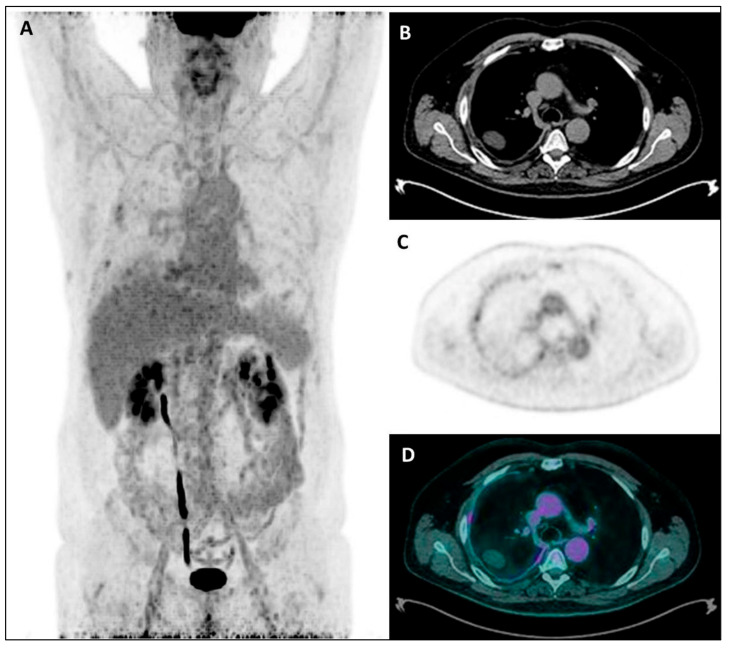
Maximum Intensity Projection—MIP (**A**), coregistered low-dose CT (**B**), ^18^F-FDG PET (**C**), and fused PET/CT images (**D**) in a male patient with diffuse right pleural thickening; only a low inhomogeneous tracer uptake was evident, along with pleural effusion. Subsequently, the patient underwent right pleurectomy with histological evidence of a diffuse epithelioid MPM.

Stated that “higher” tumor uptake was predictive of worse prognosis, the tumor uptake was quantified through SUV-based and/or volumetric semiquantitative PET parameters, with each study identifying a cut-off value for each resulting prognostic parameter, able to distinguish MPM patients with a better or worse prognosis (i.e., with tumor activity under or above the cut-off value, respectively). From the wide heterogeneity in prognostic cut-off values observed among studies, it is evident that it is not possible to establish a defined and univocal value of tumor uptake able to distinguish MPM patients with a better disease course and those with a worse prognosis. This heterogeneity in cut-off values among studies may reflect differences both in PET technical aspects, affecting the reproducibility of measured tumor activity (such as scan delay, scanner characteristics and image acquisition and reconstruction methods, beside fasting time and blood sugar level) [51], and in tumor metabolic characteristics of the study population (such as histotype and stage distribution). Regarding this latter aspect, differences in ^18^F-FDG activity have been reported among disease stages, with higher uptake values with increasing stage, and also among different MPM histological groups, with higher uptake values in sarcomatoid and biphasic subtypes than in the epithelial subtype [32,40,48], as clearly evident from Figure 2 and Figure 3. As expected according to these histological differences in metabolic activity, when a subgroup analysis was performed with respect to tumor histology, the cut-off values were lower in patients with epithelioid subtype than in those with non-epithelioid subtype, so further highlighting that the use of any cut-off should be carefully interpreted. Ideally, the identification of a more standardizable cut-off value (e.g., normalizing the tumor uptake to the physiological activity of a reference tissue such as liver or mediastinal blood-pool) would be desirable in order to introduce the PET tumor activity as a prognostic factor in the clinical practice.

Regarding the semiquantitative PET parameters investigated as prognostic predictors, SUV max was evaluated in all studies, as expected, being the commonest measure used in clinical practice to quantify tumor activity. When compared to SUVmax, the volumetric parameters (TLG and MTV) showed an overall better prognostic value. The greatest predicting performance of the volumetric parameters is reasonably explainable when considering the different “meaning” of both measure, from a functional point of view, and also the proper complex morphological appearance of MPM. Indeed, SUVmax can be considered as the tumor uptake measured in a spatially limited area of the entire tumor burden (i.e., in a single-pixel), whereas the volumetric parameters reflect the activity in the whole tumor volume, incorporating the total tumor volume and its metabolic activity. When considering that MPM presents with a diffuse, irregular, complex, and inhomogeneous tumor geometry due to a non-spherical growth pattern along the pleural surface (compared to the substantially spherical growth of the other solid tumors), MTV and TLG seem so able to better describe the intrinsic MPM heterogeneity over the single-pixel analysis provided by SUVmax, providing significant additional metabolic information [1,21,52]. From our data, a prognostic predominance of one volumetric measure over the other was not emerging; however, no sufficient data for a reliable comparison between MTV and TLG were available, due to the limited number of studies that evaluated both parameters.

**Figure 4:**
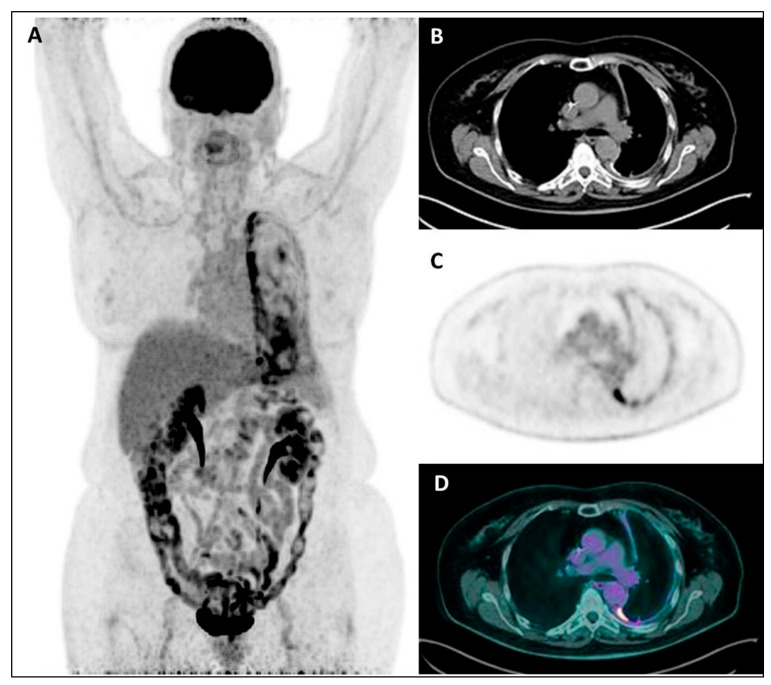
MIP (**A**), coregistered low-dose CT (**B**), ^18^F-FDG PET (**C**), and fused PET/CT images (**D**) in a female patient with recent diagnosis of MPM and submitted to talc pleurodesis: a diffuse increased^18^F-FDG uptake is evident throughout the left pleura, mainly along its mediastinal/para-vertebral side, with the CT evidence of a diffuse pleural thickening, sometimes with hyperdense spots due to talc collection.

Finally, albeit the overall prognostic value of staging ^18^F-FDG PET imaging that emerges from this review, it has to be considered that PET uptake parameters were not always confirmed as independent prognostic factors, and that a minority of studies did not find any relationship between tumor metabolic activity and survival, for a total of 9/19 included studies (almost 50%). In particular, regarding the two studies by Genestreti et al. and Özyürek et al. not reporting any prognostic role of PET imaging [32,33], both authors pointed out a limited sample size as possible explanations of their negative results. Moreover, Genestreti et al. [53] suggested a possible confounding effect of talc pleurodesis performed before PET in more than half of their patients as an additional factor limiting the results of their prognostic analysis. Indeed, it is well-known that, as result of inflammatory reaction to talc particles, an increased and intense pleural ^18^F-FDG uptake (Figure 4) occurs in sites of talc pleurodesis (that appear as a diffuse high-density pleural thickening), thus leading to an overestimation of the true MPM metabolic activity due to pleurodesis-associated inflammatory component [53,54,55]. A possible similar bias cannot be excluded in the other study by Özyürek et al. since information on patients previously treated with pleurodesis was not reported. When considering the seven studies with “partial” or “conditional” PET prognostic significance, in the three studies by Klabatsa et al. [37], Koyuncu et al. [42] and Billè et al. [44], PET uptake parameters were not or borderline significant at multivariable analysis, likely due to the limited number of patients; moreover, previous pleurodesis and use of different PET scanners were additionally reported as possible factors limiting the prognostic analysis by Billè et al. In the other three studies by Nowak et al., Hall et al. and Lim et al. [34,46,48], tumor PET uptake resulted in a weak prognostic factor in MPM patients with non-epithelioid histology (predominantly the sarcomatoid type), thus confirming the histology as a main recognized predictor of a worse prognosis. Regarding the potential co-occurrence of a confounding pleurodesis effect in these studies, no information on pleurodesis was reported by Lim et al., whereas a cohort of pleurodesis-treated patients was present in the other two studies. However, the reliability of the prognostic analysis seems not to have been influenced by pleurodesis in the study by Nowak et al., since the Authors observed that, in patients with non-sarcomatoid histology, TGV remained predictor of survival in both the pleurodesis and non-pleurodesis group. Interestingly, the same Authors developed a predictive prognostic nomogram taking into account the MPM activity overestimation induced by pleurodesis, assigning a positive correction factor (by adding points) to the patients who have not had pleurodesis. In the last study by Zucali et al. [29], the tumor metabolic changes after chemotherapy (in terms of ΔSUVmax and ΔTLG) were not associated with survival outcomes in patients treated with pleurodesis: this result supports the potential drawback of pleurodesis on the prognostic significance of PET parameters also in the post-treatment setting, due to a not-reliable evaluation of residual viable tumor. Finally, from our review data, whether pleurodesis has a different impact on the prognostic value of SUV-based or volumetric parameters at baseline is a controversial and not extensively investigated issue; although volumetric parameters (such as baseline TLG and TGV) seem to retain their prognostic role even in patients who had undergone pleurodesis [29,34], evidence on baseline SUVmax prognostic value is discordant: Zucali et al. [29] found significant correlation with PFS and OS regardless of pleurodesis, while SUVmax did not significantly predict survival in both patients’ subgroups in the study by Nowak et al. [34].

### 4.2. New Promising PET Prognostic Parameters

Beside the more widely investigated PET semiquantitative parameters, in the last decade the application of radiomics analysis to PET imaging has gained increasing interest in the oncological setting, due to the potential to improve tumor knowledge and, consequently, to contribute to personalized patients’ management. Indeed, radiomics analysis allows us to recognize, extract, and analyze a large amount of advanced quantitative features extracted from PET images, able to reflect underlying heterogeneity in biological tumor characteristics, thus providing much more information on tumor behavior than those obtained through conventional PET uptake measures [25]. In the MPM context, the recent paper by Pavic et al. [50] firstly investigated the innovative application of radiomics analysis on staging PET images with a prognostic purpose. Their study, although with a retrospective design and a small population size (training and a validation cohort of 36 patients each), suggests that a radiomics model could be a potential useful prognostic tool in MPM patients, with even better prediction performance than the commonly used SUVmax and the volumetric PET parameters. However, further analysis in a larger population and in a multi-centric setting are needed to validate their model.

Furthermore, BM metabolic pattern has been proposed as a new potential PET prognostic parameter by Ozmen et al. [45], with BM hyperactivity at visual analysis resulting associated with a poor prognosis. As suggested by the Authors, the prognostic value of BM hyperactivity seems correlated to leucocytosis, a factor in turn related with a poor prognosis in MPM patients, as supported by previous literature evidence: MPM can cause leucocytosis by simulating BM and producing granulocyte-macrophage colony stimulating factor [56,57]; increased ^18^F-FDG uptake in BM may reflect BM hypercellularity as effect of this MPM stimulation; moreover, a significant correlation between BM uptake and increased leukocyte count was observed in the same study by Ozmen et al. [45]. So, BM metabolic pattern may potentially contribute to the prognostic stratification of MPM patients as an additional qualitative PET parameter.

### 4.3. Limitations

Beside the overall small sample size and the prevalent retrospective design of the included studies, the main limitation of this review is the observed heterogeneity among studies, ranging from clinical characteristics (such as disease stages, treatment management, and mixed patients treated or untreated with previous pleurodesis) to methodological aspects (such as the analyzed PET parameters and the lesion contouring methods), all potentially affecting the reported prognostic results. Indeed, the first clinical aspect potentially impacting on PET prognostic significance is represented by disease stage, with patients presenting with limited or advanced disease differently distributed in each study and among studies, thus reasonably implying *a priori* differences in prognostic burden. In this regard, the issues related to disease stage could be minimized by PET studies focusing only on a selected population with a homogeneous clinical stage (e.g., with unresectable disease alone or exclusively eligible for surgery). In addition, the application of non-uniform treatment protocols among studies (from chemotherapy to palliative radiotherapy or trimodality treatment) may have impacted patients’ survival, potentially affecting the perceived prognostic significance of staging PET. Furthermore, the overall comparison and reproducibility of the analyzed PET semiquantitative parameters among studies is limited by an expected heterogeneity in image acquisition and reconstruction protocols (PET exams performed in different centers, with different scanners) and in lesion contouring methods. In particular, differences in tumor contouring methods may have impacted MTV and TLG prognostic analyses, being that MTV is strictly dependent from the selection of tumor VOI on PET images, and TLG, in turn, is strictly dependent on MTV by definition (being the product of SUVmean and MTV).

### 4.4. Future Perspectives

From the analyzed data, the need of conduct prospective studies, with more homogeneous and standardized methods of PET images analysis, emerges as the main future perspective. In particular, the identification of more standardized and reproducible methods of PET tumor contouring would allow a more reliable prognostic analysis of PET volumetric parameters (MTV and TLG), especially when considering that MPM is characterized by an extremely irregular tumor volume due to diffuse and asymmetric growth pattern along the pleura and frequent infiltration of thoracic structures, thus making tumor contouring not so technically easy and reproducible. Furthermore, from the recent but limited evidence of a promising role of radiomics application to PET imaging for the prognostic evaluation of MPM patients, PET radiomics analysis emerges as an appealing issue of future research in this setting. Finally, it would be interesting to investigate the prognostic role of other radiotracers (alternative to ^18^F-FDG) such as ^11^C-choline, which seems to show lower the uptake in granulomatous inflammation caused by talc pleurodesis than ^18^F-FDG, thus potentially overcoming the well-known limitations demonstrated by ^18^F-FDG in MPM patients previously treated with pleurodesis [58].

## 5. Conclusions

Our review, despite heterogeneity in several aspects among the included studies, supports the value of ^18^F-FDG PET imaging performed at staging as a prognostic predictor in MPM patients, with higher tumor uptake predictive of worse prognosis. In particular, among the PET semiquantitative parameters applied to quantify the MPM metabolic activity, the volumetric parameters (MTV and TLG) seem to be better prognostic factors than the more commonly used SUVmax, likely being able to better reflect the intrinsic heterogeneity of tumor lesions. However, it has to be considered that PET uptake parameters were not always confirmed as independent prognostic factors, especially in patients previously treated with pleurodesis and with the non-epithelioid histotype. Therefore, future prospective studies in larger and more clinically homogeneous populations, and using more standardized and reproducible methods of PET images analysis, are needed in order to validate the prognostic value of staging ^18^F-FDG PET imaging further and unequivocally in MPM patients. In this regard, a definitive consensus on the value of PET imaging as a prognostic biomarker in MPM may allow to include tumor metabolic information at staging in the prognostic stratification process, in addition to the routinely used clinico-pathological prognostic factors. And, in the perspective of personalized medicine, the additional information provided by ^18^F-FDG PET could be clinically useful for a better patient-tailored treatment planning: patients with expected less unfavorable outcomes may be addressed to intensive chemotherapy or multimodality treatment (including radical surgery), whereas those patients with expected worse prognoses may be candidates for less aggressive therapies or best supportive care, so reducing the possible toxicity of ineffective and expensive treatments and also optimizing the allocation of the economic resources of the public health system.

## Figures and Tables

**Table 1:** Main characteristics of the included studies (n = 19).

First Author	Year of Publication	Journal	Country	Study Design	Study Population with MPM	Gender (% Males)	Age (Mean ± SD or Median + Range)	Patients with Available Data for PET Prognostic Assessment	MPM Subtype (n)	Stage of Disease (n)	Clinical Indication for PET Study	Pleurodesis before PET (n)
Nowak AK [34]	2010	Clin Cancer Res	Australia	P	89	88	n.r.	89	E (69), B (13), S (7)	TNM-CT based: I (12), II (8), III (31), IV (33)	Staging	Yes (28/89)
Lee HY [35]	2010	Ann Surg Oncol	Korea	R	23	69	54 (37–61)	13	E (9), B (3), S (1)	TNM: III (12), IV (1)	Staging	No
Abe Y [38]	2012	Oncol Rep	Japan	n.r. (likely R)	31	87	67 (47–79)	23	E (14), B (6), S (4), O (2), U (5)	any stage	Diagnosis, staging	No
Kaira K [39]	2012	Eur J Cancer	Japan	R	21	86	66 (19–79)	21	E (16), B (2), S (1), D (2)	TNM: I (8), II (1), III (5), IV (7)	Staging	No
Genestreti G [32]	2012	Tech Cancer Res Treat	Italy	R	27	78	65 (54–77)	27	E (23), B (4)	TNM: I (15), II (4), III (8)	Staging	Yes (13/27)
Terada T [40]	2012	Exp Ther Med	Japan	n.r. (likely R)	47	81	65.2 ± 9.6	47	E (31), B (4), S (6), D (1), U (5)	TNM: I (9), II (10), III (9), IV (19)	Staging	n.r.
Abakay A [41]	2013	Eur Rev Med Pharm Sci	Turkey	R	177	56	55.4 ± 11.3	177	E (144), U (33)	TNM: I-II (90), III-IV (87)	Diagnosis, staging	Yes (60/177)
Klabatsa A [37]	2014	EJNMMI	UK	R	60	85	65	60	E (31), S (5), D (2), U (9), Mix (13)	AJCC: 1 (9), 2 (13), 3 (27), 4 (11)	Staging	No
Koyuncu A [42]	2015	J Cancer Res Ther	Turkey	R	60	57	53.6 ± 10.6	60	E (45), B (14), Und (1)	TNM: I (15), II (13), III (19), IV (13)	Staging	n.s.
Pinelli V [43]	2015	Respiration	Italy, USA, France	R	32	75	63 (45–74)	32	E (29), S (1), Mix (2)	TNM: I (3), II (6), III (15), IV (8)	Staging	No
Billé A [44]	2016	J Thorac Oncol	USA	R	191	77	71 (46–90)	143	E (128), B (20), S (28), U (15)	TNM: I-II (34), III (87), IV (70)	Staging	Yes (n.r.)
Ozmen O [45]	2016	Nucl Med Commun	Turkey	R	51	49	56.2 ± 11.4	51	E (30), B (13), S (1), U (7)	Locally advanced and metastatic disease	Staging	No
Zucali PA [29] *	2017	Cancer Med	Italy	R	142	66	n.r.	142	E (116), O (25), U (1)	Unresectable	Staging and treatment response	Yes (77/142)
Özyürek BA [33]	2018	Clin Resp J	Turkey	R	73	51	56.1 ± 11.4	67	E (45), B (16), U (12)	TNM: I (6), II (15), III (20), IV (32)	Staging	n.r.
Hall DO [46] *	2018	Nucl Med Commun	UK	P	73	86	73	65 with baseline PET; 54 with both baseline and post-treatment PET	E (50), B (8), S (15)	TNM: I (8), II (5), III (34), IV (26)	Staging and treatment response	Yes (27/73)
Doi H [47]	2020	Clin Lung Cancer	Japan	R	188	83	68 (31–84)	188	E (139), non-E (49)	TNM: I-II (63), III-IV (125)	Staging	n.r.
Lim JH [48]	2020	PLoS One	South Korea	R	54	76	64 (53–71)	54	E (34), B (3), S (10), U (7)	TNM: I (18), II (2), III (24), IV (10)	Staging	n.r.
Lococo F [49]	2020	Inter Card Thorac Surg	Italy	R	141	72	69 ± 9	141	E (89), B (36), S (16)	TNM: I (15), II (56), III (57), IV (13)	Staging	No
Pavic M [50]	2020	EJNMMI Res	Switzerland	R	72	89	40–76	72	E (61), B (9), S (2)	Eligible for curative surgery	Staging	n.r.

*MPM* malignant pleural mesothelioma, *SD* standard deviation, *PET* Positron Emission Tomography, *P* prospective, *R* retrospective, *n.r.* not reported, *E* epithelioid, *B* biphasic, *S* sarcomatoid, *O* other, *U* unknown, *D* desmoplastic, *Mix* mixed, *Und* undifferentiated, *non-E* non-epithelioid, *TNM* TNM cancer staging system, *CT* Computed Tomography, AJCC American Joint Committee on Cancer, *n.s.* not specified. * with PET imaging performed both at baseline (before treatment) and after treatment.

**Table 2:** Methodological aspects of ^18^F-FDG PET studies exclusively performed at baseline (n = 17, excluded studies with PET performed both before and after treatment).

First Author	PET Modality	PET/CT with c.e.	Injected Activity (MBq)	Scan Delay (Minutes p.i.)	Dual Time Imaging (Timing)	FOV	Semiquantitative PET Image Analysis	Tumor Contouring (for Volumetric Analysis)	Treatment Strategy (after PET)
Nowak AK [34]	PET	No	215/m^2^	90	No	n.r.	SUV-based + volumetric parameters	VOI on pleural lesion by iterative algorithm based on adaptive threshold	CHT, trimodality treatment, palliative RT
Lee HY [35]	PET/CT	No	n.r.	n.r.	No	n.r.	SUV-based + volumetric parameters	VOI over primary lesion using an isocontour with threshold set as liver SUVmean + 2SD	Surgery, palliative CHT
Abe Y [38]	PET/CT	No	3.7/Kg	60	Yes (delayed phase at 120 min p.i.)	n.r.	SUV-based	-	CHT, surgery + CHT, BSC
Kaira K [39]	PET/CT	No	200–250	60	No	skull top-groin	SUV-based	-	Surgery ± CHT, CHT, RT, BSC
Genestreti G [32]	PET/CT	No	5.18/Kg	50–60	No	skull-upper thighs	SUV-based	-	CHT, surgery ± CHT
Terada T [40]	PET/CT	No	n.r.	60	No	head-foot	SUV-based	-	CHT, surgery + RT
Abakay A [41]	PET/CT	Yes (oral contrast agent only)	215/m^2^	60	No	n.r.	SUV-based	-	CHT, multimodality treatment, BSC
Klabatsa A [37]	PET/CT	No	350–400	90	No	n.r.	SUV-based + volumetric parameters	VOI on tumor areas with threshold of 40% of SUVmax in 41 pts, 20% in 16 pts and 60% in 3 pts	Radical trimodality treatment, palliative therapy
Koyuncu A [42]	PET/CT	No	n.r.	n.r.	No	n.r.	SUV-based	-	Surgery, CHT, multimodality treatment, BSC
Pinelli V [43]	PET/CT	No	5.5/Kg	60	No	skull base-mid thighs	SUV-based	-	n.r.
Billé A [44]	PET	n.s.	n.r.	n.r.	No	n.r.	SUV-based	-	CHT, CHT + RT, RT
Ozmen O [45]	PET/CT	Yes (for specific cases)	370–555	60	No	vertex- proximal femur	SUV-based + volumetric parameters	VOI on pleural tumor with threshold of 40% of SUVmax	BSC, CHT, multimodality treatment, surgery
Özyürek BA [33]	PET/CT	No	370–555	60	No	skull base-high thighs	SUV-based	-	CHT, CHT + palliative RT (n = 9, 12%), surgery + CHT
Doi H [46]	PET/CT	No	4.0/Kg	60	No	head-toes	SUV-based + volumetric parameters	VOI on tumor lesions by gradient-based tumor segmentation	CHT
Lim JH [48]	PET/CT	No	5.0/Kg	60	No	n.r.	SUV-based	-	Surgery ± CHT, palliative CHT
Lococo F [49]	PET/CT	No	n.r.	n.r.	No	n.r.	SUV-based	-	Surgery (when indicated)
Pavic M [50]	PET/CT	Yes (not in all pts)	188–417	46–91	No	n.r.	SUV-based + volumetric parameters + radiomics analysis	n.s.	Surgery ± CHT

*PET* Positron Emission Tomography, *CT* Computed Tomography, *c.e.* contrast-enhancement, *p.i.* post-injection, *FOV* field of view, *n.s.* not specified, *pts* patients, *n.r.* not reported, *SUV* standardized uptake volume, *VOI* volume of interest, *SD* standard deviation, *CHT* chemotherapy, *RT* radiotherapy, *BSC* best supportive care.

**Table 3:** Methodological aspects of ^18^F-FDG PET studies performed both before and after treatment (n = 2).

First Author	PET Modality	PET/CT with c.e.	Injected Activity (MBq)	Scan Delay (Minutes p.i.)	Dual Time Imaging	FOV	Semiquantitative PET Image Analysis	Tumor Contouring (for Volumetric Analysis)	Treatment Type	PET after Treatment (Timing)
Zucali PA [29]	PET/CT (115/142), PET alone (27/142)	No	n.r.	60	No	skull base-thighs	SUV-based + volumetric parameters	VOI on metabolic tumor-related areas using a semiautomated iterative threshold-based, region-growing algorithm or a semiautomated liver-based threshold contouring	CHT (pemetrexed)	after 2 cycles of CHT
Hall DO [46] *	PET/CT	No	400	90	No	n.r.	SUV-based + volumetric parameters	VOI defined as all voxels with SUV > 2.5, within both lungs	CHT (pemetrexed + cisplatin/carboplatin); BSC	after 2 cycles of CHT

*PET* Positron Emission Tomography, *CT* Computed Tomography, *c.e.* contrast-enhancement, *p.i.* post-injection, FOV field of view, *n.r.* not reported, *SUV* standardized uptake volume, *VOI* volume of interest, *CHT* chemotherapy, *BSC* best supportive care. * PET performed at baseline in 65 pts (51 CHT, 14 BSC) and both before and after treatment in 54/65 pts (41 CHT, 13 BSC).

**Table 4:** Prognostic results of included ^18^F-FDG PET studies (n = 19).

First Author	Evaluated PET Parameters	PET Prognostic Value	Best PET Prognostic Parameters	Optimal Cut-Off of PET Prognostic Parameters	Other Prognostic Factors	Prognostic Endpoint
Nowak AK [34]	SUVmax, TGV	Yes *(in non-sarcomatoid type)*	TGV (poor prognosis for higher values)	TGV > median value (n.s.)	Sarcomatoid histology, weight loss (poor prognosis)	OS
Lee HY [35]	SUVmax, SUVmean, MTV, TLG	Yes	MTV and TLG (poor prognosis for higher values)	MTV > 250 mL; TLG > 1250	Sarcomatoid histology (poor prognosis)	TTP
Abe Y [38]	SUVmax (at early and delayed phase)	Yes	SUVmax (poor prognosis for higher values)	early SUVmax > 3.65; delayed SUVmax > 6.0	n.s.s.	OS
Kaira K [39]	SUVmax, T/M ratio (tumor SUVpeak/mediastinal SUVmean)	Yes	SUVmax and T/M ratio (poor prognosis for higher values)	SUVmax > 5.20; T/M ratio > 4.23	n.r.	OS
Genestreti G [32]	SUVmax, SUVmean	No	None	-	n.r.	OS
Terada T [40]	SUVmax	Yes	SUVmax (poor prognosis for higher values)	SUVmax > 3.5	Age > 65 years (poor prognosis)	OS
Abakay A [41]	SUVmax	Yes	SUVmax (poor prognosis for higher values)	SUVmax > 5	Male gender, KPS < 60, BSC, stage III-IV (poor prognosis)	OS
Klabatsa A [37]	SUVmax, SUVmean, SUVpeak, MTV, TLG	Yes *(borderline significance at multivariate analysis)*	MTV and TLG *(only TLG evaluated at multivariate analysis)* (poor prognosis for higher values)	MTV > 755 mL; TLG > 2914	Epithelioid histology (better prognosis)	OS
Koyuncu A [42]	SUVmax	Yes *(only at univariate analysis)*	SUVmax (poor prognosis for higher values)	SUVmax > 8	Leukocytosis, advanced stage, BSC, MAI > 1 (poor prognosis)	OS
Pinelli V [43]	SUVmax	Yes	SUVmax (poor prognosis for higher values)	SUVmax ≥ 6.1	n.r.	OS
Billé A [44]	SUVmax	Yes *(only at univariate analysis)*	SUVmax (poor prognosis for higher values)	SUV max > 8.1	Biphasic or sarcomatoid histotype, platelet count >450,000, PS 2–3 (poor prognosis)	OS
Ozmen O [45]	SUVmax, SUVmean, MTV, TLG, BM visual score (range 0–2), BM/liver ratio (BM SUVmean/liver SUVmean)	Yes	SUVmax, MTV, BM visual score (poor prognosis for higher values)	SUVmax > 8.6; MTV > 112; BM score > 2	n.s.s.	OS
Zucali PA [29] *	baseline SUVmax, baseline TLG, changes in SUVmax (ΔSUVmax) and TLG (ΔTLG) between baseline and post-treatment PET	Yes *(different significance in pts with and without pleurodesis)*	baseline SUVmax and TLG, ΔSUVmax and ΔTLG (poor prognosis for higher baseline values and for lower Δ values)	baseline SUVmax > 9.3 and baseline TLG > 534 (in pts with pleurodesis); baseline SUVmax > 6.2 and baseline TLG > 927.3 (in pts without pleurodesis); ΔSUVmax ≥ 25%; ΔTLG ≥ 30% (in pts without pleurodesis)	PS 1–2, non-epithelioid histology (poor prognosis)	PFS, OS
Özyürek BA [33]	SUVmax	No	None	-	Age ≥ 55 years (poor prognosis)	OS
Hall DO [46] *	baseline SUVmax, baseline MTV, baseline TLG, changes in SUVmax (ΔSUVmax), MTV (ΔMTV) and TLG (ΔTLG) between baseline and post-treatment PET	Yes *(significance at multivariate analysis, only if histotype was excluded)*	baseline SUVmax, baseline MTV, baseline TLG *(only SUVmax at multivariate analysis)* (poor prognosis for higher values)	SUVmax > 10.6; MTV > 460 mm^3^; TLG > 1806	Epithelioid histology (better prognosis)	PFS, OS
Doi H [47]	SUVmax, MTV, TLG	Yes	SUVmax, MTV and TLG (poor prognosis for higher values)	SUVmax ≥ 5.6; MTV ≥ 270; TLG ≥ 525 *(only TLG significant at multivariate analysis)*	Non-epitheliod histology, elevated LDH levels, NLR ≥ 5 (poor prognosis)	OS
Lim JH [48]	SUVmax	Yes *(no significance in non-epitheliod subtype)*	SUVmax (poor prognosis for higher values)	SUVmax > 10.1 (all pts); SUVmax > 8.5 (epitheliod subtype)	Epitheliod subtype, stage I–II, chemotherapy (better prognosis)	OS
Lococo F [49]	SUVmax	Yes	SUVmax (poor prognosis for higher values)	SUVmax > median SUVmax value, SUVmax > SUVmax at Q25%, SUVmax > SUVmax at Q75%	Stage II-IV, non-epithelioid histology (poor prognosis)	OS
Pavic M [50]	SUVmax, SUVmean, metabolic volume (at six different SUVmax-thresholds), radiomic features (n = 780)	Yes	3 radiomic features (prognostic power for PFS; PFS radiomics prognostic model also discriminating for OS), metabolic volume (prognostic power for OS)	n.r.	n.r.	PFS, OS

*PET* Positron Emission Tomography, *SUV* standardized uptake value, *TGV* total glycolytic volume, *MTV* metabolic tumor volume, *TLG* total lesion glycolysis, *T/M ratio* tumor to mediastinum ratio, *BM* bone marrow, *Q%* interquartile range, *KPS* Karnfsky Performance Status, *BSC* Best Supportive Care, *PS* Performance Status, *MAI* mitotic index activity, *NLR* neutrophil–lymphocyte ratio, *PFS* progression free survival, *OS* overall survival, *n.s.* not specified, *n.r.* not reported, *n.s.s.* not statistically significant. * with PET imaging performed both at baseline (before treatment) and after treatment.

## Data Availability

No new data were created or analyzed in this study. Data sharing is not applicable to this article.

## References

[1] Nickell L.T., Lichtenberger J.P., Khorashadi L., Abbott G.F., Carter B.W. (2014). Multimodality imaging for characterization, classification, and staging of malignant pleural mesothelioma. Radiographics.

[2] Opitz I. (2014). Management of malignant pleural mesothelioma—The European experience. J. Thorac. Dis..

[3] Cardinale L., Ardissone F., Gned D., Sverzellati N., Piacibello E., Veltri A. (2017). Diagnostic Imaging and workup of Malignant Pleural Mesothelioma. Acta Biomed..

[4] National Comprehensive Cancer Network Guidelines in Oncology: Malignant Pleural Mesothelioma. https://www.nccn.org/professionals/physician_gls/pdf/mpm.pdf.

[5] Flores R.M., Routledge T., Seshan V.E., Dycoco J., Zakowski M., Hirth Y., Rusch V.W. (2008). The impact of lymph node station on survival in 348 patients with surgically resected malignant pleural mesothelioma: Implications for revision of the American Joint Committee on Cancer staging system. J. Thorac. Cardiovasc. Surg..

[6] Tsao A.S., Wistuba I., Roth J.A., Kindler H.L. (2009). Malignant pleural mesothelioma. J. Clin. Oncol..

[7] Lococo F. (2021). Malignant Pleural Mesothelioma: Time Is Running Out. J. Clin. Med..

[8] de Gooijer C.J., Baas P., Burgers J.A. (2018). Current chemotherapy strategies in malignant pleural mesothelioma. Transl. Lung Cancer Res..

[9] Katzman D., Sterman D.H. (2018). Updates in the diagnosis and treatment of malignant pleural mesothelioma. Curr. Opin. Pulm. Med..

[10] Nakano T. (2008). Current therapies for malignant pleural mesothelioma. Environ. Health Prev. Med..

[11] Scherpereel A., Astoul P., Baas P., Berghmans T., Clayson H., de Vuyst P., Dienemann H., Galateau-Salle F., Hennequin C., Hillerdal G. (2010). Guidelines of the European Respiratory Society and the European Society of Thoracic Surgeons for the management of malignant pleural mesothelioma. Eur. Respir. J..

[12] Curran D., Sahmoud T., Therasse P., van Meerbeeck J., Postmus P.E., Giaccone G. (1998). Prognostic factors in patients with pleural mesothelioma: The European Organization for Research and Treatment of Cancer experience. J. Clin. Oncol..

[13] Herndon J.E., Green M.R., Chahinian A.P., Corson J.M., Suzuki Y., Vogelzang N.J. (1998). Factors predictive of survival among 337 patients with mesothelioma treated between 1984 and 1994 by the Cancer and Leukemia Group B. Chest.

[14] Imperatori A., Castiglioni M., Mortara L., Nardecchia E., Rotolo N. (2013). The challenge of prognostic markers in pleural mesothelioma. J. Thorac. Dis..

[15] Ambrogi V., Mineo T.C., Multidisciplinary Tor Vergata University Study Group for Malignant Pleural Mesothelioma (2012). Clinical and biologic prognostic factors in malignant pleural mesothelioma. Thorac. Cancer.

[16] Blyth K.G., Murphy D.J. (2018). Progress and challenges in Mesothelioma: From bench to bedside. Respir. Med..

[17] Frauenfelder T., Kestenholz P., Hunziker R., Nguyen T.D., Fries M., Veit-Haibach P., Husmann L., Stahel R., Weder W., Opitz I. (2015). Use of computed tomography and positron emission tomography/computed tomography for staging of local extent in patients with malignant pleural mesothelioma. J. Comput. Assist. Tomogr..

[18] Rusch V.W. (1996). A proposed new international TNM staging system for malignant pleural mesothelioma from the International Mesothelioma Interest Group. Lung Cancer.

[19] Flores R.M., Akhurst T., Gonen M., Larson S.M., Rusch V.W. (2003). Positron emission tomography defines metastatic disease but not locoregional disease in patients with malignant pleural mesothelioma. J. Thorac. Cardiovasc. Surg..

[20] Kruse M., Sherry S.J., Paidpally V., Mercier G., Subramaniam R.M. (2013). FDG PET/CT in the management of primary pleural tumors and pleural metastases. AJR Am. J. Roentgenol..

[21] Kitajima K., Doi H., Kuribayashi K. (2016). Present and future roles of FDG-PET/CT imaging in the management of malignant pleural mesothelioma. Jpn. J. Radiol..

[22] Fuccio C., Spinapolice E.G., Ferretti A., Castellucci P., Marzola M.C., Trifiro G., Rubello D. (2013). ^18^F-FDG-PET/CT in malignant mesothelioma. Biomed. Pharmacother..

[23] Sharif S., Zahid I., Routledge T., Scarci M. (2011). Does positron emission tomography offer prognostic information in malignant pleural mesothelioma?. Interact. Cardiovasc. Thorac. Surg..

[24] Aide N., Lasnon C., Veit-Haibach P., Sera T., Sattler B., Boellaard R. (2017). EANM/EARL harmonization strategies in PET quantification: From daily practice to multicentre oncological studies. Eur. J. Nucl. Med. Mol. Imaging.

[25] Hirata K., Tamaki N. (2021). Quantitative FDG PET Assessment for Oncology Therapy. Cancers.

[26] Incerti E., Broggi S., Fodor A., Cuzzocrea M., Samanes Gajate A.M., Mapelli P., Fiorino C., Dell’Oca I., Pasetti M., Cattaneo M. (2018). FDG PET-derived parameters as prognostic tool in progressive malignant pleural mesothelioma treated patients. Eur. J. Nucl. Med. Mol. Imaging.

[27] Tsutani Y., Takuwa T., Miyata Y., Fukuoka K., Hasegawa S., Nakano T., Okada M. (2013). Prognostic significance of metabolic response by positron emission tomography after neoadjuvant chemotherapy for resectable malignant pleural mesothelioma. Ann. Oncol..

[28] Kitajima K., Maruyama M., Minami T., Yokoi T., Kuribayashi K., Kijima T., Hashimoto M., Hasegawa S., Yamakado K. (2020). Comparison of modified Response Evaluation Criteria in Solid Tumors, European Organization for Research and Treatment of Cancer criteria, and PET Response Criteria in Solid Tumors for evaluation of tumor response to chemotherapy and prognosis prediction in patients with unresectable malignant pleural mesothelioma. Nucl. Med. Commun..

[29] Zucali P.A., Lopci E., Ceresoli G.L., Giordano L., Perrino M., Ciocia G., Gianoncelli L., Lorenzi E., Simonelli M., De Vincenzo F. (2017). Prognostic and predictive role of [^18^F]fluorodeoxyglucose positron emission tomography (FDG-PET) in patients with unresectable malignant pleural mesothelioma (MPM) treated with up-front pemetrexed-based chemotherapy. Cancer Med..

[30] Lopci E., Zucali P.A., Ceresoli G.L., Perrino M., Giordano L., Gianoncelli L., Lorenzi E., Gemelli M., Santoro A., Chiti A. (2015). Quantitative analyses at baseline and interim PET evaluation for response assessment and outcome definition in patients with malignant pleural mesothelioma. Eur. J. Nucl. Med. Mol. Imaging.

[31] Ceresoli G.L., Chiti A., Zucali P.A., Rodari M., Lutman R.F., Salamina S., Incarbone M., Alloisio M., Santoro A. (2006). Early response evaluation in malignant pleural mesothelioma by positron emission tomography with [^18^F]fluorodeoxyglucose. J. Clin. Oncol..

[32] Genestreti G., Moretti A., Piciucchi S., Tiseo M., Bersanelli M., Scarlattei M., Scarpi E., Dubini A., Matteucci F., Sanna S. (2012). Prognostic value of ^18^F-FDG standard uptake value by integrated PET/CT in the staging of malignant pleural mesothelioma. Technol. Cancer Res. Treat..

[33] Ozyurek B.A., Ozmen O., Ozdemirel T.S., Erdogan Y., Kaplan B., Kaplan T. (2018). Relation between neutrophil/lymphocyte ratio and primary tumor metabolic activity in patients with malign pleural mesothelioma. Clin. Respir. J..

[34] Nowak A.K., Francis R.J., Phillips M.J., Millward M.J., van der Schaaf A.A., Boucek J., Musk A.W., McCoy M.J., Segal A., Robins P. (2010). A novel prognostic model for malignant mesothelioma incorporating quantitative FDG-PET imaging with clinical parameters. Clin. Cancer Res..

[35] Lee H.Y., Hyun S.H., Lee K.S., Kim B.T., Kim J., Shim Y.M., Ahn M.J., Kim T.S., Yi C.A., Chung M.J. (2010). Volume-based parameter of ^18^F-FDG PET/CT in malignant pleural mesothelioma: Prediction of therapeutic response and prognostic implications. Ann. Surg. Oncol..

[36] Veit-Haibach P., Schaefer N.G., Steinert H.C., Soyka J.D., Seifert B., Stahel R.A. (2010). Combined FDG-PET/CT in response evaluation of malignant pleural mesothelioma. Lung Cancer.

[37] Klabatsa A., Chicklore S., Barrington S.F., Goh V., Lang-Lazdunski L., Cook G.J. (2014). The association of ^18^F-FDG PET/CT parameters with survival in malignant pleural mesothelioma. Eur. J. Nucl. Med. Mol. Imaging.

[38] Abe Y., Tamura K., Sakata I., Ishida J., Ozeki Y., Tamura A., Uematsu K., Sakai H., Goya T., Kanazawa M. (2012). Clinical implications of ^18^F-fluorodeoxyglucose positron emission tomography/computed tomography at delayed phase for diagnosis and prognosis of malignant pleural mesothelioma. Oncol. Rep..

[39] Kaira K., Serizawa M., Koh Y., Takahashi T., Hanaoka H., Oriuchi N., Endo M., Kondo H., Nakajima T., Yamamoto N. (2012). Relationship between ^18^F-FDG uptake on positron emission tomography and molecular biology in malignant pleural mesothelioma. Eur. J. Cancer.

[40] Terada T., Tabata C., Tabata R., Okuwa H., Kanemura S., Shibata E., Nakano T. (2012). Clinical utility of 18-fluorodeoxyglucose positron emission tomography/computed tomography in malignant pleural mesothelioma. Exp. Ther. Med..

[41] Abakay A., Komek H., Abakay O., Palanci Y., Ekici F., Tekbas G., Tanrikulu A.C. (2013). Relationship between 18 FDG PET-CT findings and the survival of 177 patients with malignant pleural mesothelioma. Eur. Rev. Med. Pharmacol. Sci..

[42] Koyuncu A., Koksal D., Ozmen O., Demirag F., Bayiz H., Aydogdu K., Berkoglu M. (2015). Prognostic factors in malignant pleural mesothelioma: A retrospective study of 60 Turkish patients. J. Cancer Res. Ther..

[43] Pinelli V., Roca E., Lucchini S., Laroumagne S., Loundou A., Dutau H., Maldonado F., Astoul P. (2015). Positron Emission Tomography/Computed Tomography for the Pleural Staging of Malignant Pleural Mesothelioma: How Accurate Is It?. Respiration.

[44] Bille A., Krug L.M., Woo K.M., Rusch V.W., Zauderer M.G. (2016). Contemporary Analysis of Prognostic Factors in Patients with Unresectable Malignant Pleural Mesothelioma. J. Thorac. Oncol..

[45] Ozmen O., Koyuncu A., Koksal D., Tatci E., Alagoz E., Demirag F., Gokcek A., Arslan N. (2016). The potential value of volume-based quantitative PET parameters and increased bone marrow uptake for the prediction of survival in patients with malignant pleural mesothelioma. Nucl Med. Commun..

[46] Hall D.O., Hooper C.E., Searle J., Darby M., White P., Harvey J.E., Braybrooke J.P., Maskell N.A., Masani V., Lyburn I.D. (2018). ^18^F-Fluorodeoxyglucose PET/CT and dynamic contrast-enhanced MRI as imaging biomarkers in malignant pleural mesothelioma. Nucl. Med. Commun..

[47] Doi H., Kuribayashi K., Kitajima K., Yamakado K., Kijima T. (2020). Development of a Novel Prognostic Risk Classification System for Malignant Pleural Mesothelioma. Clin. Lung Cancer.

[48] Lim J.H., Choi J.Y., Im Y., Yoo H., Jhun B.W., Jeong B.H., Park H.Y., Lee K., Kim H., Kwon O.J. (2020). Prognostic value of SUVmax on ^18^F-fluorodeoxyglucose PET/CT scan in patients with malignant pleural mesothelioma. PLoS ONE.

[49] Lococo F., Rena O., Torricelli F., Filice A., Rapicetta C., Boldorini R., Paci M., Versari A. (2020). ^18^F-fluorodeoxyglucose positron emission tomography in malignant pleural mesothelioma: Diagnostic and prognostic performance and its correlation to pathological results. Interact. Cardiovasc. Thorac. Surg..

[50] Pavic M., Bogowicz M., Kraft J., Vuong D., Mayinger M., Kroeze S.G.C., Friess M., Frauenfelder T., Andratschke N., Huellner M. (2020). FDG PET versus CT radiomics to predict outcome in malignant pleural mesothelioma patients. EJNMMI Res..

[51] Keyes J.W. (1995). SUV: Standard uptake or silly useless value?. J. Nucl. Med..

[52] Bianco A., Valente T., De Rimini M.L., Sica G., Fiorelli A. (2018). Clinical diagnosis of malignant pleural mesothelioma. J. Thorac. Dis..

[53] Genestreti G., Moretti A., Piciucchi S., Giovannini N., Galassi R., Scarpi E., Burgio M.A., Amadori D., Sanna S., Poletti V. (2012). FDG PET/CT Response Evaluation in Malignant Pleural Mesothelioma Patients Treated with Talc Pleurodesis and Chemotherapy. J. Cancer.

[54] Nguyen N.C., Tran I., Hueser C.N., Oliver D., Farghaly H.R., Osman M.M. (2009). F-18 FDG PET/CT characterization of talc pleurodesis-induced pleural changes over time: A retrospective study. Clin. Nucl. Med..

[55] Kwek B.H., Aquino S.L., Fischman A.J. (2004). Fluorodeoxyglucose positron emission tomography and CT after talc pleurodesis. Chest.

[56] Yoshimoto A., Kasahara K., Saito K., Fujimura M., Nakao S. (2005). Granulocyte colony-stimulating factor-producing malignant pleural mesothelioma with the expression of other cytokines. Int. J. Clin. Oncol..

[57] Edwards J.G., Abrams K.R., Leverment J.N., Spyt T.J., Waller D.A., O’Byrne K.J. (2000). Prognostic factors for malignant mesothelioma in 142 patients: Validation of CALGB and EORTC prognostic scoring systems. Thorax.

[58] Kitajima K., Nakamichi T., Hasegawa S., Kuribayashi K., Yamakado K. (2018). Fluorodeoxyglucose versus Choline Positron Emission Tomography/Computed Tomography Response Evaluation in Two Malignant Pleural Mesothelioma Patients Treated with Talc Pleurodesis and Neoadjuvant Chemotherapy. Cureus.

